# CD90 Marks a Mesenchymal Program in Human Thymic Epithelial Cells *In Vitro* and *In Vivo*

**DOI:** 10.3389/fimmu.2022.846281

**Published:** 2022-03-16

**Authors:** Shicheng Sun, Jacky Y. Li, Hieu T. Nim, Adam Piers, Mirana Ramialison, Enzo R. Porrello, Igor E. Konstantinov, Andrew G. Elefanty, Edouard G. Stanley

**Affiliations:** ^1^ Murdoch Children’s Research Institute, The Royal Children’s Hospital, Parkville, VIC, Australia; ^2^ Faculty of Medicine, Dentistry and Health Sciences, University of Melbourne, Parkville, VIC, Australia; ^3^ The Novo Nordisk Foundation Center for Stem Cell Medicine (reNEW), Murdoch Children’s Research Institute, Parkville, VIC, Australia; ^4^ Australian Regenerative Medicine Institute and Systems Biology Institute Australia, Monash University, Clayton, VIC, Australia; ^5^ Melbourne Centre for Cardiovascular Genomics and Regenerative Medicine, Royal Children’s Hospital, Melbourne, VIC, Australia; ^6^ Department of Cardiac Surgery, Royal Children’s Hospital, Melbourne, VIC, Australia; ^7^ Department of Anatomy and Developmental Biology, Monash University, Clayton, VIC, Australia

**Keywords:** human thymic epithelial cells, epithelial and mesenchymal components, primary cells culture, pluripotent stem cell differentiation, CD90/Thy1, cell identity

## Abstract

Thymic epithelium is critical for the structural integrity of the thymus and for T cell development. Within the fully formed thymus, large numbers of hematopoietic cells shape the thymic epithelium into a scaffold-like structure which bears little similarity to classical epithelial layers, such as those observed in the skin, intestine or pancreas. Here, we show that human thymic epithelial cells (TECs) possess an epithelial identity that also incorporates the expression of mesenchymal cell associated genes, whose expression levels vary between medullary and cortical TECs (m/cTECs). Using pluripotent stem cell (PSC) differentiation systems, we identified a unique population of cells that co-expressed the master TEC transcription factor *FOXN1*, as well as the epithelial associated marker EPCAM and the mesenchymal associated gene CD90. Using the same serum free culture conditions, we also observed co-expression of EPCAM and CD90 on cultured TECs derived from neonatal human thymus *in vitro*. Single cell RNA-sequencing revealed these cultured TECs possessed an immature mTEC phenotype and expressed epithelial and mesenchymal associated genes, such as *EPCAM*, *CLDN4*, *CD90* and *COL1A1*. Importantly, flow cytometry and single cell RNA-sequencing analysis further confirmed the presence of an EPCAM+CD90+ population in the CD45- fraction of neonatal human thymic stromal cells *in vivo*. Using the human thymus cell atlas, we found that cTECs displayed more pronounced mesenchymal characteristics than mTECs during embryonic development. Collectively, these results suggest human TECs possess a hybrid gene expression program comprising both epithelial and mesenchymal elements, and provide a basis for the further exploration of thymus development from primary tissues and from the *in vitro* differentiation of PSCs.

## Introduction

The thymus is a haematopoietic organ where T cells develop, and central tolerance is established. The capacity to regenerate a functional analogue of this organ *in vitro* would provide an accessible and tractable experimental platform to study T cell development, and to gain a greater understanding of how tolerance is established and how it is undermined, the latter leading to autoimmunity (1, 2). Previously, we and others have used human pluripotent stem cells (PSCs) to generate thymic endodermal progenitor cells that, in principle, have the potential to differentiate into functional cortical or medullary thymic epithelial cells (c/mTECs) capable of supporting T cell differentiation (3–6). However, to date, the most advanced differentiation protocols have not been able to generate functional TECs from hPSCs *in vitro*. Instead, functional differentiation has only been achieved following transplantation into immunodeficient mice (3, 4, 6). A confounding factor in recreating the thymic epithelium *de novo* is the number of cell types that have a role in its genesis, coupled with insufficient knowledge concerning the origins and characteristics of TECs.

In distinction to other epithelial organs, functional development of the thymic epithelium relies on the influx of hematopoietic cells, which rapidly enlarge the thymus (7–9). Along with this, the thymic epithelium undergoes a drastic morphological change; the epithelial primordium is transformed from a tight cluster of epithelial cells into a scaffold-like structure that is interspersed with large numbers of T cell progenitors (10, 11). These changes coincide with the specification of cortical and medullary thymic epithelial cells (cTEC and mTECs), both of which are thought to derive from a common TEC progenitor (12–15). cTECs and mTECs are distinguished from each other by their location, functionality, and repertoire of gene expression. Classically, the pattern of cytokeratin expression has been used to distinguish mTECs and cTECs; TEC progenitors expressed both keratin 5 (KRT5) and KRT8, whose expression is subsequently restricted to mTECs and cTECs, respectively (16). Additionally, mTECs are also distinguished from cTECs by the former’s expression of KRT14. Apart from keratin expression, cTECs and mTECs also develop distinct epithelial phenotypes. Immunofluorescence analysis shows that human thymic primordium at embryonic week 7 expresses high levels of EPCAM, a marker that is retained in mTECs at embryonic week 15 but substantially downregulated in cTECs (7). Similarly, the tight junction-forming proteins claudin 3 (CLDN3) and CLDN4 are highly enriched in mTECs (17). These observations indicate that TEC specification is coupled with morphological and molecular changes in typical epithelial characteristics, some of which may be important in the process of TEC differentiation from hPSCs.

Forkhead box protein N1 (FOXN1) is a master transcription factor that plays a critical role in the development of thymic epithelial cells (18). FOXN1 is detected mid-week 6 of human development, with its expression restricted to a site within the third pharyngeal pouch that marks the presumptive thymus primordia, in distinction to the presumptive parathyroid which is marked by GCM2 (glial cells missing transcription factor 2); both tissues develop from the third pharyngeal pouch (7, 19, 20). However, studies using Foxn1 deficient mice indicate that initial thymic commitment is Foxn1 independent, but that Foxn1 is required for specification of cTEC and mTEC from TEC progenitors (18) and Foxn1-null thymic primordium is unable to support hematopoietic colonization (21). In the context of hPSC differentiation *in vitro*, currently available methods direct differentiation to a stage where *FOXN1* expression is detectable, but cells fail to undergo further functional commitment (3, 5, 6). We previously generated *FOXN1*:GFP hPSC reporter lines that are a valuable tool for further dissecting the molecular regulation of human TEC development and for the isolation and examination of FOXN1+ cells (5).

In this study, we examined the characteristics of hPSC-derived thymic progenitors and neonatal thymic epithelial cells cultured under the same serum free conditions. Experiments using single cell RNA-sequencing confirmed that CD90 (also known as THY1), a gene often associated with mesenchymal cells, is expressed by human TECs and that this expression reflects a broader underlying mesenchymal gene expression program. Indeed, we show FOXN1+ TECs and TEC progenitors isolated from *in vivo* and *in vitro* sources co-expressed EPCAM and CD90. Further analysis revealed that human TECs expressed a cohort of mesenchymal markers, suggesting that TECs acquired an identity with characteristics of both epithelial and mesenchymal cell types. These findings provide biological insight into human thymic epithelial cell identity and a basis to further explore thymic development from pluripotent stem cells.

## Materials and Methods

### Thymic Endoderm Differentiation From Human PSC

Work related to pluripotent stem cell lines was conducted in accordance with RCH Human Research Ethics Committee 33001A. Two *FOXN1*:GFP human PSC reporter lines (MEL1 and HES3) were used to generate FOXN1+ epithelial cells following our previously published protocol with modifications (5). Human PSCs were cultured with a standard E8 medium (Gibco)-based feeder-free cell culture system as described defined (22). At day 0, cells were harvested and deposited into each well (3 × 10^3^cells/well) of a 96-well round-bottom nonadherent plate (Nunc) and briefly centrifuged to promote cell aggregation in to embryoid bodies (EBs). Differentiation was set up using our new chemically defined serum- and albumin-free (CD-SAF) medium (**Table 1**), supplemented with 100 ng/ml Activin A for 5 days for endoderm induction. On day 5, medium was replaced with only the CD-SAF medium without additional supplements. On day 7, EBs were transferred to gelatin-coated (0.1%) 96-well flat-bottom adherent plates (BD Falcon). From day 14, medium was supplemented with 40 ng ml^−1^ human keratinocyte growth factor (KGF; Peprotech, 100-19). Analyses of these cultures were performed between day 30 to day 60. In all instances PSC cultures and differentiations were maintained at 37°C, in a 5% CO_2_/air environment.

**Table 1:** Chemically defined serum- and albumin-free cell culture medium.

Items	Stock	Final	For 500 ml	REF/Cat#	Supplier
ITS-X (E)	100X	1X	5ml	777ITS032	InVitria
Polyvinyl alcohol	10%	0.1%	5ml	P8136-1KG	SIGMA
Methyl cellulose	10%	0.1%	5ml	M7027-250G	SIGMA
AA2P (L-Ascorbic acid 2-phosphate)	10 mg/L	50 ug/ml	2.5 ml	A8960-5G	SIGMA
Glutamax	100 X	1X	5 ml	35050-061	Gibco
NEAA	100 X	1X	5 ml	11140-050	Gibco
Lipid Concentrates		1/500	1ml	11905-031 (100ML)	Gibco
Embryo MAX Nucleosides	100X	1X	5ml	ES008-D	Millipore
Pen/Strep		1/200	2.5ml	15140-122 (100ML)	Gibco
IMDM/F12 media 1:1 mix			Up to 500 ml		Gibco

### Neonatal Human Thymus Tissue Collection

Neonatal thymus tissues were obtained from Melbourne Heart Tissue Bank at The Royal Children’s Hospital (RCH) from pediatric patients in accordance with the policies and ethics of RCH and Melbourne Children’s Heart Tissue Bank. Samples were collected from infants, younger than one-year-old, who were diagnosed with congenital heart defects and underwent cardiac surgery. Tissue collection for research purposes was obtained under the human ethics approval (HREC 38192) following informed consent by a parent or guardian.

### Thymic Stromal Cell Collection

Neonatal thymus tissue was mechanically disrupted to release thymocytes. Briefly, the thymus tissue was cut into pieces of approximately 0.5 cm^3^, and then, using the plunger of a 20-ml disposable syringe, pressed against the membrane of a 40-µm cell strainer sitting in a sterile 6-cm tissue-culture plate with DME medium. Thymocytes were flushed through the membrane using cold DMEM medium. This process was repeated 4 times to dislodge blood cells. Then, the thymus stroma was minced into small pieces using surgical scissors. The minced thymic tissues were transferred to a Falcon tube and further dissociated using Collagenase Type 1 (2 ng/ml in IMDM medium, Worthington-biochem) at 37 °C for 4-5 hours. The cell suspension was then centrifuged, and collagenase buffer aspirated. The cell pellet was washed with cold PBS. Thymic cells were finally resuspended in the CD-SAF medium containing 10 ng/ml KGF and 10 μM Rock inhibitor Y-27632 (Stemcell Technologies, 72304) and plated onto Geltrex pre-coated 6-well cell culture plates. For analysis of fresh human thymic stromal cells, the cell solution was then passed through the cell-strainer cap of a FACS tube to ensure a single-cell suspension.

### Culture of Neonatal Thymus-Derived Stromal Cells

During the first two weeks, the CD-SAF medium containing 5 ng/ml KGF was replenished every 3 days. At one week after plating, epithelial colonies consisting of human thymic cells with a polygonal shape emerged. After two weeks, the cell culture was passaged at a 1:2 ratio. Briefly, cells were washed once with PBS and then dissociated using prewarmed TrypLE (1X, ThermoFisher) for 5 minutes at 37°C. Then, a 1-ml Gilson pipette was used to physically dissociate the cells by repeatedly pipetting the cell solution. The cell solution was then diluted in PBS, to neutralize TrypLE, transferred to a 15 ml Falcon tube, and centrifuged for 3 minutes at 4C. Following removal of the supernatant the cell pellet was resuspended in the CD-SAF medium containing 5 ng/ml KGF and 10 μM Rock inhibitor Y-27632 and the cells then transferred to fresh Geltrex pre-coated plates. The CD-SAF medium containing 5 ng/ml KGF was replenished every 3 days. From this passage, human thymic epithelial cell cultures were passaged weekly at a 1:2 splitting ratio.

### Flow Cytometry Analysis and Cell Sorting

Characterization of adherent cultures required dissociation into single cells by incubation with prewarmed TrypLE-select™ at 37 °C. Incubation time varied with the type of cell culture; 5 minutes for neonatal human thymus-derived TEC cultures and 10-15 minutes for and PSC-derived FOXN1+ cultures. Conjugated monoclonal mouse anti-human antibodies: CD104-APC (1:50, clone 422325, Invitrogen), EPCAM-PeCy7 (1:200, clone 12c2, BioLegend), EPCAM-BV421(1:50, clone 9C4, BioLegend), CD90-PE (1:100, clone 5e10, Biolegend), CD90-BV421 (1:50, clone 5e10, Biolegend) were diluted in FACS wash buffer (PBS supplemented with 5% fetal bovine serum) and incubated with cells for 20 minutes on ice. The cell suspension was washed twice with FACS wash solution to remove unbound antibodies and resuspended in FACS wash solution containing 1 μg/ml propidium iodide. Cell surface staining was examined using a Becton Dickenson (BD) LSRFortessa Cell Analyzer. Flow cytometry data were analyzed using the FlowLogic program (7.2.1, DataNova). Alternatively, cell purification was performed using a BD FACSaria FUSION or Influx cell sorter based on cell surface staining or the expression of a fluorescent reporter. Cells were collected using a 5ml FACS tube containing 0.5ml cold fetal calf serum.

### RNA Sequencing

RNA was isolated using the ISOLATEII RNA micro-Kit (Bioline, BIO-52075) as described by the manufacturer. Library preparation and sequencing was performed by sequencing facility at the Victorian Clinical Genetic Services (VCGS) in Melbourne. Library was sequenced on by the Illumina Novaseq-6000 system for 20 million reads per sample. STAR aligner was used to map bulk sequencing data with the GRCh38-3.0.0 genome. Sequencing data was processed using the RNAsik pipeline (23). The mapped count files generated were uploaded onto Degust (https://degust.erc.monash.edu) to perform differential gene analysis using the Voom method (24). Data related to genes deemed to be statistically significant were exported as a count matrix for further analyses with R version 3.6.1 to generate heatmaps and other visualizations. Pathway analyses and gene enrichment of selected genes was completed *via* Metascape analysis (25).

### Single Cell RNA-Sequencing

Single cell suspension samples were prepared at 1,000,000 cells/ml with viability at approximate 90%. RNA extraction and library preparation were performed by the Victorian Clinical Genetics Service following 10x Genomics’s Cell Preparation Guide (https://www.10xgenomics.com). Sequencing was performed with the Illumina Novaseq-6000 system with a target of 50,000 read depth for 6000 cells. FASTQ files generated from sequencing were used with the 10x genomics software cell ranger (version 6.0.2) to map reads to the human reference genome version GRCH38-3.0.0. This generated an output including the information related to each cell’s barcoding, matrix including counts and features information.

Standard single cell RNA sequencing analysis was completed on RStudio (R version 3.6.1) with the Seurat package (version 4.0.1). Data preprocessing was completed for quality control purposes. Cells with less than 200 genes and more than 10,000 genes were excluded along with those that expressed more than 25% mitochondria, more than 30% ribosomal and more than 1.5% mitoribosome expression. Following normalization (NormalizeData, scale factor 10,000), integration across samples was completed with canonical correlation analysis (CCA). All genes within the original matrix were used to identify anchors that were then used for integration. This allowed for the identification of thymic epithelial cell identities, as we integrated our monolayer cells from primary human thymus samples (HTS) and hPSC-derived FOXN1+ cells with the published human thymus cell atlas  (26). Data of uncultured primary cells was derived from the epithelial cell subset instead of total thymic cells, which was annotated by Park et al. in their original study. This annotated matrix specific to epithelial cells excludes conventional mesenchymal cells/fibroblasts, which is publicly available through Zenodo.org (https://zenodo.org/record/3711134#.YgBTaC_aGCR). This annotated thymic epithelial cell matrix was used for analysis in **Figure 3** for a direct comparison with *in vitro* derived human TECs and in **Figure 5** for identification of TECs expressing CD90. The integrated matrix of gene expression across samples was used for data scaling and cell cycle genes were regressed within the same step. The number of dimensions (n=20) was selected along with a resolution of 0.5 for cell clustering. FindMarkers, FeaturePlot and DotPlot analysis of cluster-specific genes was used to identify cluster identity. Rmagic (version 2.0.3) was used to uncover epithelial and mesenchyme signatures in **Figure 4** and **Figure S4** within the integrated samples with default parameters (27). Standard parameters within the package were used.

### Data Availability

RNA-sequencing data is available in the public GEO data repository with the identification number GSE196005.

## Results

### PSC Differentiation Identifies CD90 Expression as a Marker of FOXN1+ Thymic Endodermal Progenitor Cells

**Figure 1:**
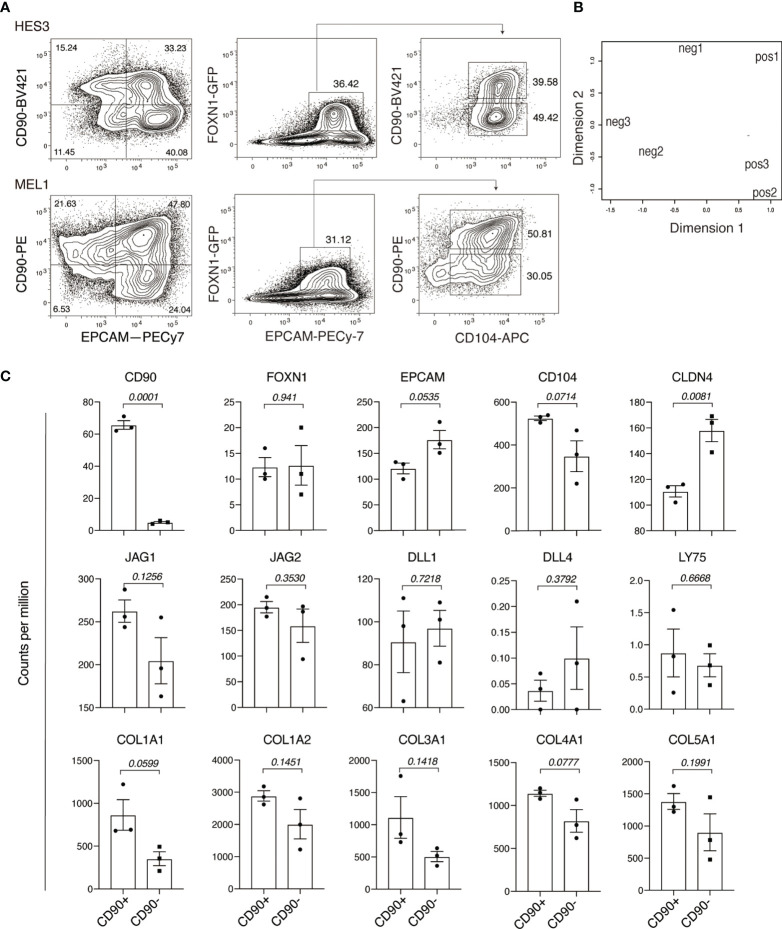
Human pluripotent stem cell differentiation identifies a FOXN+EPCAM+CD90+ population. **(A)** Flow cytometry analysis of PSC-derived endodermal cultures for the expression of CD90, EPCAM, *FOXN1*:GFP and CD104. Data shows representative results of two independent PSC lines: HES3 and MEL1. **(B)** RNA-sequencing analysis of GFP+EPCAM+CD90+ (pos) and GFP+EPCAM+CD90- (neg) fractions showing the relationships between individual samples representing each population in a multi-dimensional scaling (MDS) plot. **(C)** Histogram representation of the expression of thymic associated genes, NOTCH ligand genes and collagens. The Y axis shows expression in counts per million (CPM) for GFP+EPCAM+CD90+ (CD90+) and GFP+EPCAM+CD90- (CD90-) populations. p values are of the comparison between CD90 positive and CD90 negative populations. Data is shown as the mean+/- SEM for biological replicates n = 3. Statistical significance was calculated using an unpaired t test, p values are indicated for each individual graph.

We previously described *FOXN1*:GFP PSC lines that enable the identification and purification of endodermal epithelial cells committed to the TEC lineage (5). Studies with these lines showed that *FOXN1*:GFP+ cells were marked by co-expression of EPCAM and CD104 (known as ITGB4). We have reproduced this result with feeder-free hESC cultures using a newly optimized PSC differentiation medium, chemically defined, and serum- and albumin-free (designated CD-SAF medium), based on our previously developed APEL medium (28). Previous analyses showed that these cultures contained cells that were PDGFRα+ and EPCAM-, suggestive of a fibroblastic or mesenchymal cell type. In order to further investigate this possibility, we examined cells for the expression of another widely used mesenchymal associated marker, CD90 (29). Unexpectedly, in addition to the presence of an EPCAM-CD90+ population, these cultures also contained FOXN1+ cells that co-expressed EPCAM and CD90. Indeed, FOXN1+ TEC progenitor cells could be divided into two sub-populations based on CD90 expression (**Figure 1A**).

To investigate the significance CD90 expression within the FOXN1+ cell population, we characterized cells representing the FONX1+CD90+ and FOXN1+CD90- fractions using RNA-sequencing. Principle component analysis showed that these fractions clustered separately, indicative of underlying differences in their gene expression profiles (**Figure 1B**). Further analysis also confirmed that surface expression of CD90 was faithfully reflected in the expression of *CD90* at the transcript level (**Figure 1C**). Consistent with flow cytometry analysis, *FOXN1* expression was comparable between the CD90+ and CD90- populations. Although not reaching statistical significance, the expression levels *EPCAM* and *CD104* suggested a possible difference in the expression of these two markers between the FONX1+CD90+ and FOXN1+CD90- populations. Indeed, we found that CD90- cells showed significantly higher levels of *CLDN4*, a gene whose expression is associated with mTECs (17). Conversely, neither of the two populations expressed the cTEC marker *LY75* (CD205) (30). We also found that both FONX1+CD90+ and FOXN1+CD90- populations expressed the NOTCH ligands *JAG1*, *JAG2*, *DLL1*, but not *DLL4*. Interestingly, this analysis also showed a consistent trend of increased expression of individual collagen genes in the CD90+ fraction, such *COL1A1*, which is also a well-established marker of cells undergoing mesenchymal transition from epithelial cells (**Figure 1C**) (31). Collectively, these results indicate that CD90 marked a subpopulation of hESC-derived FOXN1+EPCAM+ TEC progenitor cells.

### Derivation of Epithelial Cells From Neonatal Human Thymus in Chemically Defined Medium

Given that PSC-derived TEC progenitors generated *in vitro* represent an artificial system, we sought to examine the expression EPCAM, CD90, CD104 and FOXN1 on epithelial cells cultured from dissociated neonatal human thymus. In these experiments, we used the same CD-SAF medium employed above in order to aid the direct comparison between PSC and primary tissue-derived cell types. Thymic stromal cells were isolated from pediatric thymus tissue by physical separation followed by collagenase treatment. The cell suspension was subsequently seeded onto Geltrex-coated tissue culture plates in CD-SAF medium supplemented with 10 ng/ml keratinocyte growth factor (KGF).

**Figure 2:**
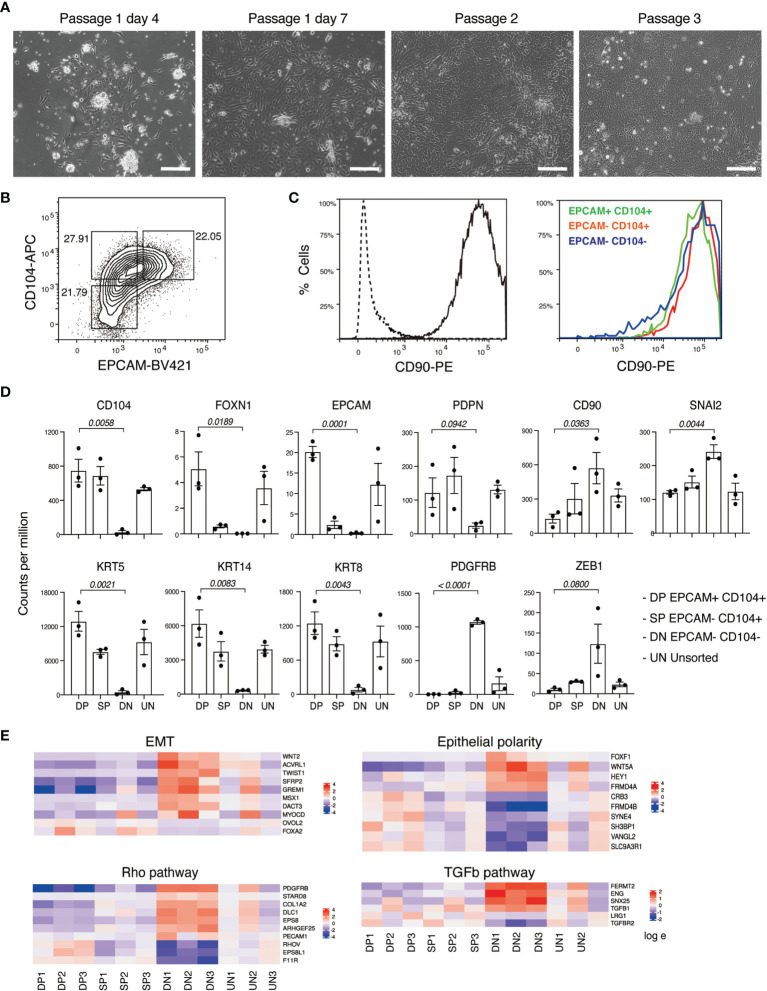
Derivation and characterization of neonatal human thymus-derived monolayer cell cultures. **(A)** Bright field images showing freshly derived and passaged monolayer adherent cells from neonatal human thymus. Sale bar, 100 mm. **(B)** Flow cytometry analysis for CD104 and EPCAM expression on neonatal thymus-derived monolayer cell cultures. **(C)** Histogram representation of flow cytometry analysis for CD90 expression of neonatal thymus-derived monolayer cell culture (left) and of indicated fractions in **(B)** identified based on CD104 and EPCAM expression. **(D)** Histogram representation of gene expression levels of TEC associated genes including keratins in each of the four indicated populations measured in counts per million (from RNA-sequencing analysis). The indicated p values relate to the comparison of the EPCAM+ CD104+ double positive (DP) with EPCAM- CD104- double negative (DN) populations. Data shown in +/- with biological replicates n=3. Statistical significance was calculated with an unpaired t test **(E)**. Heatmap representation of the log fold change in the expression levels of genes found to be statistically significant (p value < 0.05) related to EMT, epithelial polarity, Rho and TGFβ signaling in the four indicated fractions. DP, EPCAM+ CD104+ double positive; DN, EPCAM- CD104- double negative; SP, EPCAM-CD104+ double positive; UN, unsorted sample.

One week later, we observed the emergence of colonies that comprised cells displaying a polygonal epithelial morphology (**Figure 2A**). These cells could be passaged weekly for more than 4 weeks. Over this time a single confluent well at day 0 routinely gave rise to 6 confluent wells by day 30. We further characterized *in vitro* cultured neonatal thymus derived epithelial cells by flow cytometry and RNA sequencing analysis. Flow cytometry analysis showed that pediatric thymus-derived stromal cells expressed the TEC-associated surface markers EPCAM and CD104, and that this expression was maintained over at least 3 consecutive passages (**Figure 2B**). Interestingly, nearly all cells expressed CD90, and its expression levels were not substantially different between subpopulations separated on the basis of EPCAM and CD104 expression (**Figure 2C**).

### Neonatal Thymus Derived Epithelial Cells Express CD90 and EMT-Associated Genes

Next, we performed RNA-sequencing analysis of the above subpopulations based on their expression of EPCAM and CD104 (**Figure 2B** and **Figure S1A**). At the global level, principal component analysis and differentially expressed gene analysis indicated that each sorted fraction, representing EPCAM+CD104+, EPCAM-CD104- and EPCAM-CD104+ cells, was clearly separated from the other (**Figures S1B, C**). The expression levels of *EPCAM* and *CD104* transcripts correlated with the surface marker profile of each fraction, validating the integrity of the sorting strategy (**Figure 2D** and **Figure S1A**). Notably, *FOXN1* transcripts were effectively restricted to EPCAM+CD104+ cells. Moreover, the expression pattern of the recently identified TEC-associated marker *PDPN* (32) was similar to that of *CD104*. We found that EPCAM-CD104- double negative cells did not express the TEC-associated keratin genes *KRT5*, *KRT8* or *KRT14* whereas EPCAM+CD104+ double positive cells expressed the highest levels of these three keratins. Conversely, EPCAM-CD104- cells exclusively expressed the mesenchymal cell marker *PDGFRB*, suggesting a phenotype of conventional fibroblasts. In addition, *CD90* expression appeared to be progressively upregulated across the series EPCAM+CD104+, EPCAM-CD104+, EPCAM-CD104-, which pattern was also observed with the expression of mesenchymal associated transcription factors *SNAI2* and *ZEB1* (**Figure 2D**). Given the association of CD90 expression with mesenchymal cells, and the inverse correlation between its expression and that of EPCAM, we explicitly examined the expression of genes associated with the mesenchymal transition and cell polarity in the three distinct cell fractions. This analysis suggested a gradation of gene expression whereby the EPCAM-CD104- cells possessed stronger mesenchymal characteristics than the EPCAM-CD104+ and EPCAM+CD104+ populations (**Figure 2E** and **Figure S1D**). Conversely, EPCAM+CD104+ cells expressed higher levels of genes associated with epithelial polarity than the other two populations. In general, mesenchymal characteristics were corelated with the levels of *CD90* transcript (**Figure 2E**). Collectively, these results suggest that expression of CD90 marks a mesenchymal like program within neonatal thymus-derived epithelial cells, the degree of which is less pronounced than the conventional CD90+EPCAM-CD104- mesenchymal-like population associated with these cultures.

### Single Cell RNA-Sequencing for Neonatal Human Thymus-Derived Epithelial Cells *In Vitro*

**Figure 3:**
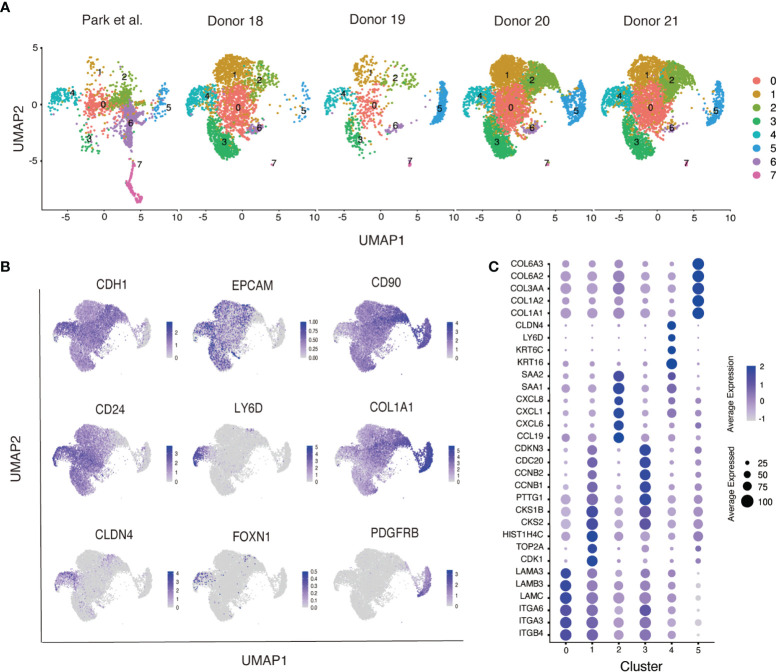
Singe cell RNA-sequencing of neonatal human thymus-derived monolayer cell cultures. **(A)** Uniform Manifold Approximation and Projection (UMAP) analysis of single cell RNA-sequencing analysis showing cells from the four donors (Donor 18-21) integrated with primary neonatal human thymic epithelial cells from the Human Thymus Cell Atlas (26). Samples were separated by their original sample identity and grouped by cell type clustering. **(B)** Feature plots of key genes expressed in monolayer cultured neonatal human thymic cells. **(C)** Dot plot representation of cluster specific genes in the monolayer cultured neonatal human thymic cells. Distinct classes of integrins, laminins, cell cycling, chemokines and their ligands (CXCLs and CCLs), mTEC associated keratins, collagens, and serum amyloids (SAAs) are identified specific to respective clusters. Color intensity in each dot represents the average expression. Dot size represents the percentage of cells expressing that gene in its respective cluster.

To examine the potential relationship between distinct populations of neonatal thymus derived epithelial cells we further characterized these cultures by single cell RNA-sequencing analysis. In this experiment, we analyzed samples that were derived from four independent donors (Donor 18-21), which contained varying proportions of the subpopulations marked by expression of EPCAM and CD104 (**Figure S2A**). Using conical correlation analysis (CCA function in Seurat), we integrated our cells with primary neonatal TECs from the human thymus cell atlas, the latter serving as a reference for the identities of cultured TECs (26) (**Figure S2B**). Therefore, this analysis allowed a direct comparison between the *in vitro* cultured thymic stromal cells and primary neonatal human TECs. Our results showed that *in vitro* cultured cells from the four donors showed a similar pattern of cell clustering to each other and also contained a limited number of populations that were present in primary neonatal human TECs (**Figure 3A**).

Our results showed that *in vitro* cultured cells contained two basic cell types distributed across 8 clusters (**Figure S2B**); clusters 0-4 and 6-7 were epithelial cells that expressed E-cadherin (*CDH1*) and cluster 5 comprised mesenchymal cells that expressed platelet derived growth factor receptor beta (*PDGFRB*) (**Figure 3B**). *EPCAM* was weakly expressed in the *CDH1*+ epithelial population whilst nearly all cells expressed *CD90* (**Figure 3B**). The *CDH1*+ epithelial population also expressed TEC-associated keratins (*KRT5, KRT8 and KRT14*), that were rare in the *PDGFRB*+ mesenchymal population (**Figure S2C**), consistent with our results of RNA-sequencing of the sorted cell fractions (**Figure 2D**). Although overall, cultured cells expressed the *KRT8* transcript, expression of other classical cTEC markers *LY75* and *PBSM11* was not detected. This contrasts with primary TECs, where expression of both *LY75* and *PBSM11* was clearly detected in cluster 7, suggesting a cTEC cluster that was effectively absent from cultured TEC populations (**Figure S2C**). Cluster 6 contained cells that expressed *CLDN4* and *AIRE*, suggestive of a mature mTEC phenotype (**Figure S2C**). As with the cTEC population encompassed by cluster 7, the mature mTEC population contained within cluster 6 was also only present in primary cell populations (**Figure S2C**). However, *CLDN4*+ cells were also found in cluster 4, which contained both primary and cultured cells. Within this cluster we detected a rare LY6D+ population that contained FOXN1+ cells (**Figure 3B**). In addition to *CLDN4*, cluster 4 was enriched for the expression of the mTEC associated gene *CD24*, a marker that was also broadly expressed at lower levels throughout the culture cell populations (**Figure 3B**). Collectively, our analysis suggested that cultured thymus derived epithelial cells possessed a phenotype that resembled immature mTECs.

In addition to cluster 4 marked by *CLDN4* and *CD24*, primary and cultured thymus derived cells also contributed to cluster 0 (**Figure 3C**). Differentially expressed gene analysis showed that this cluster contained cells that expressed genes encoding integrins, including *ITGB4*, *ITGA3* and *ITGA6*, and laminins, including *LAMB3* and *LAMA3*. Few primary TECs were found in clusters 1, 2 and 3, suggesting these clusters comprised cells that were generated under our specific culture conditions. Differentially expressed gene analysis and gene ontology analysis showed cluster 1 and 3 were enriched with genes responsible for cell cycle and division, whereases cluster 4 expressed genes associated with immune functions, such as chemokines receptor/ligands (*CCL19*, *CXCL6*, *CXCL1* and *CXCL8*). Collectively, our flow cytometry and RNA-sequencing analysis revealed that neonatal TECs cultured *in vitro* contained a heterogeneous collection of cells that expressed genes that are associated with epithelial cell state and a restricted set of mTEC genes.

### Single Cell RNA-Sequencing Analysis Identifies a Mesenchymal Signature of Human Thymic Epithelial Cells

To further explore the relationship between epithelial and mesenchymal gene signatures within *in vivo* human TECs populations, we independently analyzed epithelial cell subpopulations in the human thymus cell atlas (26). This single cell RNA-sequencing dataset includes human TECs from different development time points ranging from week 7 embryos to 40-year-old adults. Following the standard Seurat pipeline, our analysis revealed the co-clustering of early- and mid-embryonic development time points, whereas neonate, adolescent and adult cells formed individual clusters (**Figures S3A, B**). In particular, medullary and cortical compartments within these cells were further specified using classical cTEC markers (*LY75* and *PSMB11*) and mTEC markers (*KRT14*, *CLDN4*, *AIRE* and *FEZF2*) (**Figures S3C, D**) (16, 17, 30, 33). As with the original analysis performed in Python by 26, we also identified cTEC and mTEC subpopulations at different developmental time points (**Figure S3D**).

**Figure 4:**
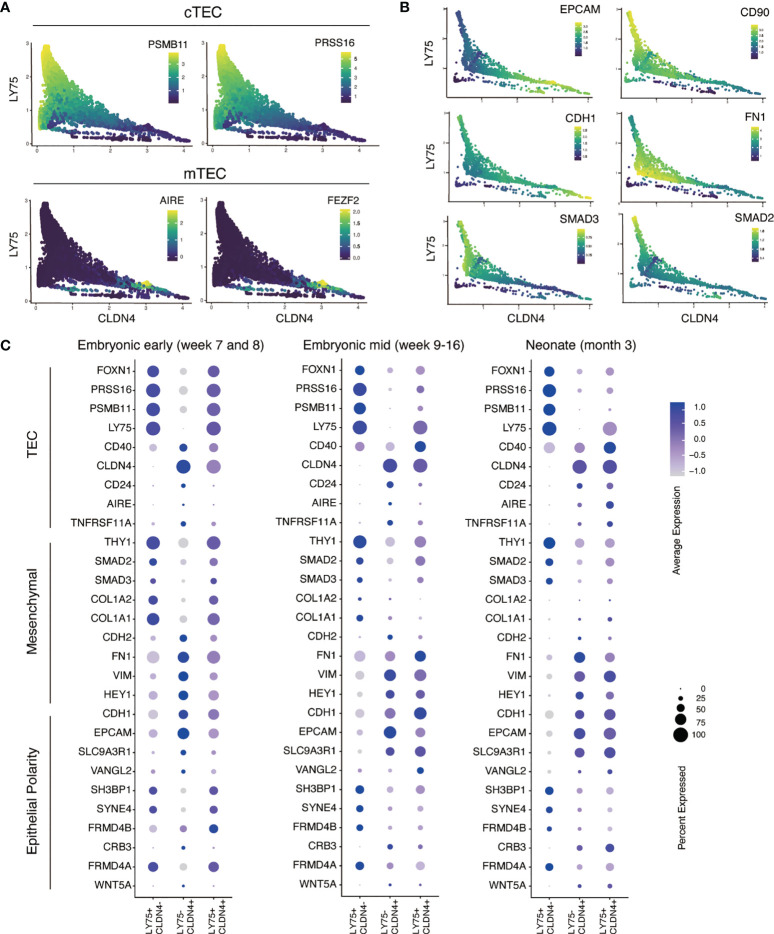
Single cell RNA-sequencing analysis of epithelial and mesenchymal gene expression in primary human cTECs and mTECs. **(A)** Validation of MAGIC program for gene-gene association analysis with established cTEC and mTEC markers and functional genes. Scatter plots show the distribution of cells associated with CLDN4 expression representing mTECs (X axis) and LY74 representing cTECs (Y axis). Color intensity represents the level of gene expression as indicated by color key. **(B)** Scatter plots showing the MAGIC imputed values calculated for epithelial and mesenchymal gene expression associated with cTECs and mTEC genes in neonate human TECs. **(C)** Dot plot representation of the expression epithelial, mesenchymal and TEC genes in primary human TECs reported in the human thymic cell atlas (26). Color intensity in each dot represents the average expression. Dot size represents the percentage of cells expressing that gene in its respective cluster.

Next, we investigated the association of epithelial/mesenchymal gene expression signatures with human TECs using the MAGIC program, an imputation analysis tool that has been validated to study gene-gene interactions in the epithelial-to-mesenchymal transition (EMT) (27). First, we tested our analysis by examining the relationship between the expression of known functional TEC genes with markers of medullary (*CLDN4*) and cortical (*LY75*) identity (**Figure 4A**). This analysis showed that the cTEC functional genes, *PSMB11* and *PRSS16*, were enriched in the *LY75*-high population, whilst the mTEC functional genes, *AIRE* and *FEZF2*, were restricted to a *CLDN4*-high population. These results support the application of MAGIC as a valid tool for studying gene-gene interactions in the context of thymic epithelial cell identity within this dataset.

We further applied MAGIC to investigate TEC subpopulations and their relationship to epithelial and mesenchymal programs. We focused on three TEC development stages: pre-hematopoietic colonization (early embryonic) (**Figure S4A**), hematopoietic colonization (mid embryonic) (**Figure S4B**) and fully functional thymus (neonate) (**Figure 4B**). We examined multiple epithelial and mesenchymal genes and their association to cTECs and mTECs, defined by the expression of *LY75* and *CLDN4*, respectively. Our results showed that *EPCAM* and *CDH1* (E-cadherin), established epithelial cell surface markers, were upregulated in concert with increasing expression of *CLDN4* (**Figure 4B**). Conversely, the EMT-related genes *FN1* (fibronectin) and *CD90* were strongly associated with *LY7*5. Similarly, we found that the intracellular signaling molecules, *SMAD2* and *SMAD3*, were associated with *LY75*, suggesting a potential activity of the TGFβ cell signaling pathway in cTECs, a pathway known to be important in the generation of mesenchymal phenotypes from epithelial cells (34). Interestingly, TECs that expressed both *LY75* and *CLDN4* transcripts, potentially representing cells at the cortical medullary junction, possessed a hybrid expression pattern that contained both epithelial and mesenchymal associated genes (**Figure 4C**). These results highlight that cTECs and mTECs show distinct patterns of epithelial and mesenchymal gene expression, with the former having a more pronounced mesenchymal gene signature, including expression of CD90.

**Figure 5:**
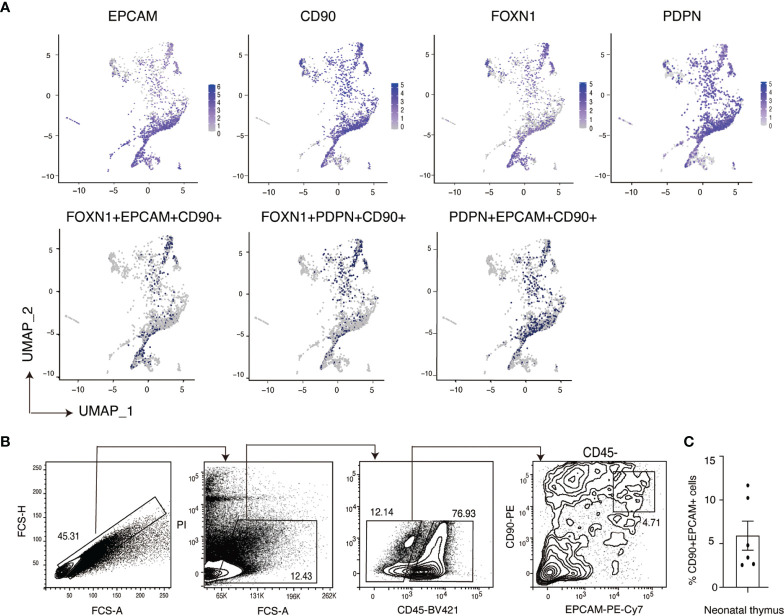
Identification of EPCAM+CD90+ cells in primary neonatal human TECs. **(A)** Feature plot representation of single cell RNA-sequencing analysis of human neonatal human thymic epithelial cells expressing EPCAM, CD90, FOXN1 and PDPN separately and triple positive populations expressing these genes. **(B)** Representative flow cytometry plots (Donor 19) showing the expression of EPCAM and CD90 on primary neonatal human TECs. Non-thymocyte cells (middle) are enriched by an FCS-A gate (left). Thymic stromal cells (right) are enriched from the CD45- population. **(C)** Quantification summary of the frequency of CD90+ EPCAM+ double positive cells within the CD45- thymic stromal cell population of six human thymus donors. Data is shown as the mean +/- SEM. Flow cytometry results contributing to the data pots in **(B)** are shown in **Figure S5**.

In neonatal thymus, we also detected TECs that were triple positive for EPCAM, CD90 and FOXN1 (**Figure 5A**). Single cell RNA-sequencing analysis also showed the expression of *CD90* on TECs marked by *PDPN* (32). To confirm the expression of CD90 on human TECs, we analyzed neonatal thymus samples by flow cytometry. This analysis showed that approximately 5% of the CD45- non-hematopoietic thymic stromal population were CD90+EPCAM+ double positive cells (**Figure 5B**). This result was reproduced with neonatal human thymus samples from another five independent donors (**Figure S5**). Collectively, these results confirm the expression of CD90 on a subset of human TECs and suggest that human TECs exhibit a hybrid program of gene expression that has elements of mesenchymal and epithelial cell states.

### Mouse Thymic Epithelial Cells Did Not Express CD90 (Thy1)

Since CD90 was originally identified as a specific thymocyte antigen in the mouse, we also surveyed its expression by re-analyzing a mouse thymus single cell RNA-sequencing dataset (35) (**Figure S6**). We annotated mouse thymus cell identities including TECs (*Epcam, Foxn1, Ly75* and *Cldn4*), thymocytes (*Ptprc* and *Cd3e*), myeloid cells (*Ptprc*, *Mpo*, *Cd52*, *Itgam* (CD11b)), endothelial cells (*Pecam1* and *Cdh5*), conventional mesenchymal cells (*Pdgfra* and *Col1a2*) and parathyroid cells (*Gcm2*). We found that Epcam was a faithful marker for TECs, covering almost all Foxn1+ cells in the developing thymus. Within the TEC population, *Ly75* and *Cldn4* further subdivided epithelial cells into two major compartments as cTECs and mTECs. Interestingly, unlike the human, mouse TECs rarely expressed *Thy1* (CD90) and its expression was restricted to thymocytes and a small fraction of mesenchymal cells. These results suggest a phenotypic difference of TECs between human and mouse, highlighting at least one mechanism of TEC-thymocyte interactions that is not conversed between the two species.

## Discussion

In this study, we provide evidence that human TECs possess a hybrid gene expression program comprising both epithelial and mesenchymal genes. Flow cytometry and gene expression profiling analysis identified CD90 as a potential marker of cells that possessed a mesenchymal-like program within cTEC populations. By developing a chemically defined serum free culture medium, we were able to derive TEC-like cells from human ESCs and neonatal human thymus, both of which provide a platform for studying TEC biology.

Our analysis showed expression of CD90 in multiple contexts of human TECs, including freshly isolated and cultured neonatal TECs (**Figures 2C**, **5B**), as well as human ESC-derived FOXN1+ TEC progenitor cells (**Figure 1A**). CD90, originally called THY1 (thymocyte differentiation antigen 1), is a specific surface marker of developing thymocytes in the mouse but not in the human (36). More broadly, CD90 has been shown to mark mesenchymal cells, including those that have transitioned from an epithelial state. In the latter case, cells of this phenotype also upregulate genes encoding extracellular matrix proteins, such as COL1A1 (34). In our PSC derived thymic endodermal cultures, we found that CD90 was expressed on EPCAM- non-epithelial cells, a cell type we had previously shown also expressed the mesenchymal marker PDGFRA (5). Unexpectedly, in the current study, we identified a distinct FOXN1+EPCAM+CD104+ population that expressed CD90 (**Figure 1A**). We confirmed the existence of a similar population of cells within neonatal TECs by both single cell RNA-sequencing analysis and flow cytometry (**Figure 5**). Indeed, analyses performed in the 1970s and 1980s suggested CD90 expression on cultured TECs and on the human cortical epithelium (37–39). More recently, Campinoti et al. identified the expression of CD90 on various TEC populations and also speculated that this expression may indicate an underlying hybrid epithelial/mesenchymal phenotype (40). Complementing our work showing expression of CD104 (integrin beta 4 subunit), Campinoti et al. found that CD104’s sole pairing subunit CD49f (integrin alpha 6 subunit) was also expressed in human TECs. Their study taken in conjunction with our own work strongly argues that human TECs have an unconventional epithelial phenotype that includes mesenchymal-like characteristics. Indeed, our single cell transcriptomic analysis revealed that CD90 is more strongly associated with cTECs throughout embryonic development (**Figures 4B** and **Figure S4**), suggesting that the outer cortical structure involves cells with a more pronounced mesenchymal signature. It is tempting to speculate that the open scaffold structure of the cortex, that results from its dramatic enlargement following hematopoietic colonization (7), could play a causative role in driving the mesenchymal characteristics of resident TEC populations. By contrast, medullary TECs, which are subject to a structurally distinct environment with fewer interceding blood cells, possess a more epithelial-like phenotype. These observations may give additional clues to further understanding in thymic epithelial cell identities and could provide novel insights into culturing techniques to derive human TECs *in vitro*.

The above observations suggest that cTECs and mTECs expressed distinct genes associated with different epithelial cell identities. Cell identity is defined by location and the repertoire of expressed genes - two parameters that directly determine a cell’s functionality. cTECs and mTECs in a fully functional thymus are believed to originate from the same bipotent progenitor population (12–14). During development, changes in location can precipitate changes in gene expression, and thus modulate cell identity. Epithelial cells frequently form a continuous layer, known as tissue epithelium, in which each cell is tightly connected to neighboring cells to create a defined axis and cell polarity (41). These cells share common characteristics in gene expression, such as the expression of EPCAM, by which they maintain the epithelium integrity and epithelial cell identity. However, our results suggest that epithelial identity may be influenced by changes in location that accompany organ morphogenesis and acquisition of functionality. The expression of mesenchymal associated genes in cTECs suggests that epithelial cells that participate in development-regulated migration events can adopt mesenchymal-like characteristics, and thus, possess a less pronounced epithelial phenotype (**Figure 4**). This phenomenon has been documented in other developmental systems; during liver development, EPCAM expression is maintained during hepatoblast differentiation towards cholangiocytes but is lost as cells form hepatocytes (42). Additionally, kidney epithelial cells also retain some mesenchymal characteristics that are potentially carried over from their immediate mesenchyme precursors that condense over the ureteric bud during renal development (43). Interestingly, it has been speculated that the retention of these characteristics might make kidney epithelial cells prone to undergoing an EMT under stress or inflammatory conditions (44, 45). These examples suggest that transitions across different epithelial and mesenchymal states is a common property of many cell systems and may present opportunities to manipulate cell phenotypes to create new cell types with new identities and functionality.

To minimize the influence of serum on the epithelial cell phenotype, we developed a chemically defined serum-free medium that permitted derivation of epithelial cells from the neonatal thymus and pluripotent stem cells (**Figures 1**, **2**). This chemically defined medium avoids inherent risks of reproducibility often associated with serum products, including fetal calf serum and human serum sourced albumin (46, 47). As such, this medium provides a stable cell culture system to identify downstream biological consequences of defined treatments or experimental conditions. As indicated by the expression of *CLDN4* and *CD24* but not *LY75*, we found that using KGF as a sole growth factor promoted an immature mTEC like phenotype in PSC-derived FOXN1+ cells and in neonatal thymus-derived monolayer epithelial cells (**Figures 1C**, **3B**). Nevertheless, these mTEC-like cells did not express functional genes, such as *AIRE* and *FEZF2*. We speculate that this might be due to the lack of certain components in the culture that can drive functional differentiation of mTECs, such as lymphoid hematopoietic cells and conventional thymic mesenchymal cells (48, 49). As such, the addition of hematopoietic factors such as stem cell factor and interleukin 7, mesenchyme factors including fibroblast factor FGF2, as well as PDGF, may support the growth or survival of these auxiliary cell types. In addition, expression of functional TECs genes of *AIRE* and *FEZF2* might be induced from our immature mTECs by additional factors to activate key pathways, such as the lymphotoxin and the RANK signals (1, 50). Therefore, future experiments could examine these variables to promote the assembly of an artificial human thymus organ culture.

## Data Availability Statement

The data presented in the study are deposited in the GEO repository, accession number GSE196005. Data is publicly released and accessible via this identifier: https://www.ncbi.nlm.nih.gov/geo/query/acc.cgi?acc=GSE196005.

## Ethics Statement

The studies involving human participants were reviewed and approved by the Royal Children’s Hospital Human Research Ethics Committee 33001A for work related to human pluripotent stem cell lines. Human thymus tissue collection for research purposes was obtained under the human ethics approval (HREC 38192) at the Royal Children’s Hospital following informed consent by a parent or guardian. The patients/participants provided their written informed consent to participate in this study.

## Author Contributions

Conceptualization, SS and ES. Methodology SS, JL, and ES. Investigation, SS and JL. Reagents Acquisition AP, EP, and IK. Formal analysis, SS, JL, and HTN. Supervision, AE, ES, and MR. Writing-original draft, SS and ES. Writing-review & editing, all authors. Funding acquisition, AE and ES. All authors contributed to the article and approved the submitted version.

## Funding

This study was funded by the National Health & Medical Research Council of Australia through research fellowships awarded to AE (GNT1117596) and ES (GNT1079004) and project grants awarded to AE and ES (GNT1129861, GNT1138717, GNT1123277), and by the Stafford Fox Medical Research Foundation. MR is funded by an NHMRC Ideas Grant (APP1180905). Additional infrastructure funding to the Murdoch Children’s Research Institute was provided by the Australian Government National Health and Medical Research Council Independent Research Institute Infrastructure Support Scheme and the Victorian Government’s Operational Infrastructure Support Program. The Novo Nordisk Foundation *Center for Stem Cell Medicine* is supported by Novo Nordisk Foundation grants (NNF21CC0073729). The Melbourne Centre for Cardiovascular Genomics and Regenerative Medicine (CardioRegen) and the Melbourne Children’s Heart Tissue Bank (MCHTB) are funded by the RCH Foundation, Shine On Foundation and the Loti and Victor Smorgon Family Foundation.

## Conflict of Interest

The authors declare that the research was conducted in the absence of any commercial or financial relationships that could be construed as a potential conflict of interest.

## Publisher’s Note

All claims expressed in this article are solely those of the authors and do not necessarily represent those of their affiliated organizations, or those of the publisher, the editors and the reviewers. Any product that may be evaluated in this article, or claim that may be made by its manufacturer, is not guaranteed or endorsed by the publisher.
