# Supplementary material for: CD90 Marks a Mesenchymal Program in Human Thymic Epithelial Cells In Vitro and In Vivo
Source: Front Immunol. 2022 Mar 16;13:846281. doi: 10.3389/fimmu.2022.846281 (PMC8966383; doi:10.3389/fimmu.2022.846281)
Supplement: Supplementary file 1 [file DataSheet_1.pdf]

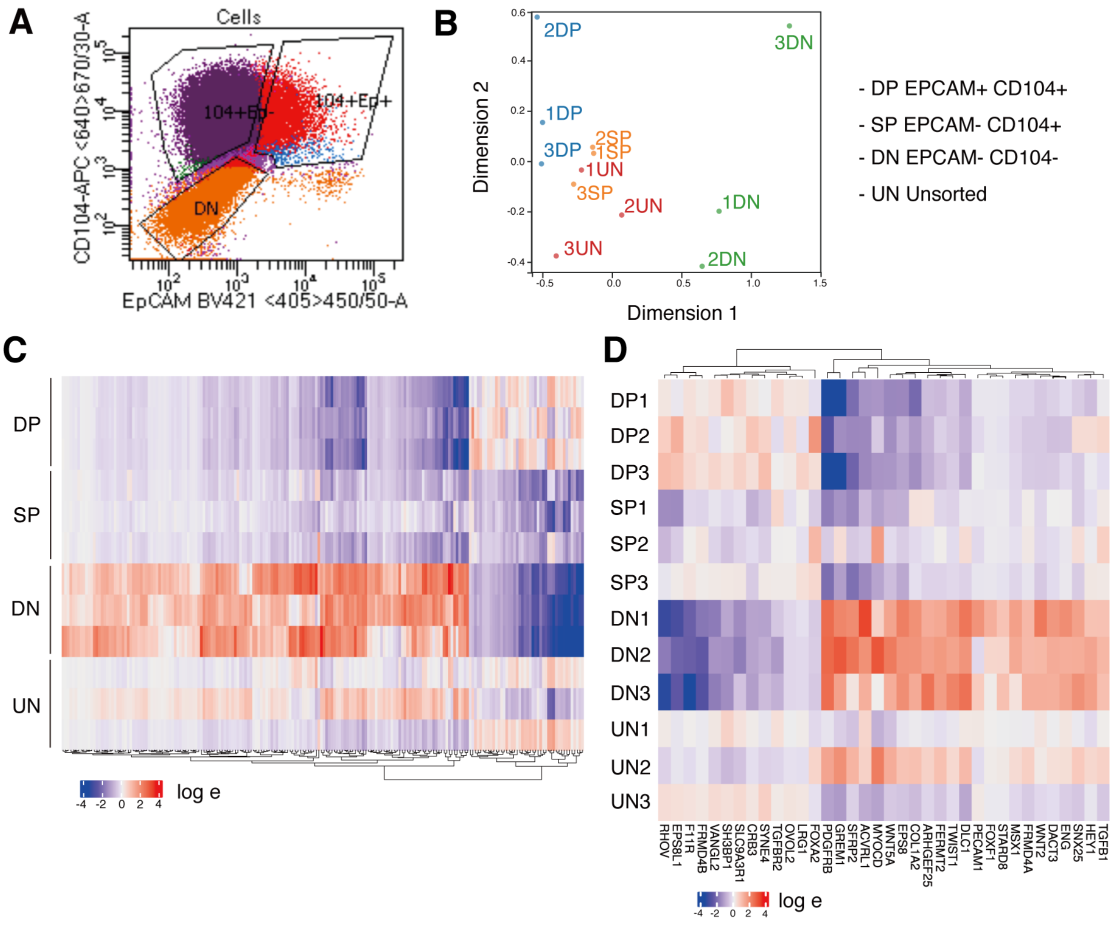

Figure S1. RNA-sequencing analysis of neonatal human thymus-derived monolayer cells cultures.

(A) Gating strategy of cell sorting for the indicated populations from the monolayer neonatal thymic cell cultures. Sorted fractions include a CD104-EPCAM- double negative (DN) population, a CD104+EPCAM- single positive (SP) population and a CD104+EPCAM+ double positive (DP) population. **(B)** Multi-dimensional scaling (MDS) plot of a total of 12 samples including three biological replicates representing populations described in (A) and three replicates of an unsorted population for each experiment. **(C)** Heatmap analysis of the top 200 differentially expressed genes in terms of log e fold change of the indicated populations. **(D)** Heatmap summary of the expression of key genes associated with the EMT program and epithelial polarity, corresponding to those shown in Figure 2E.

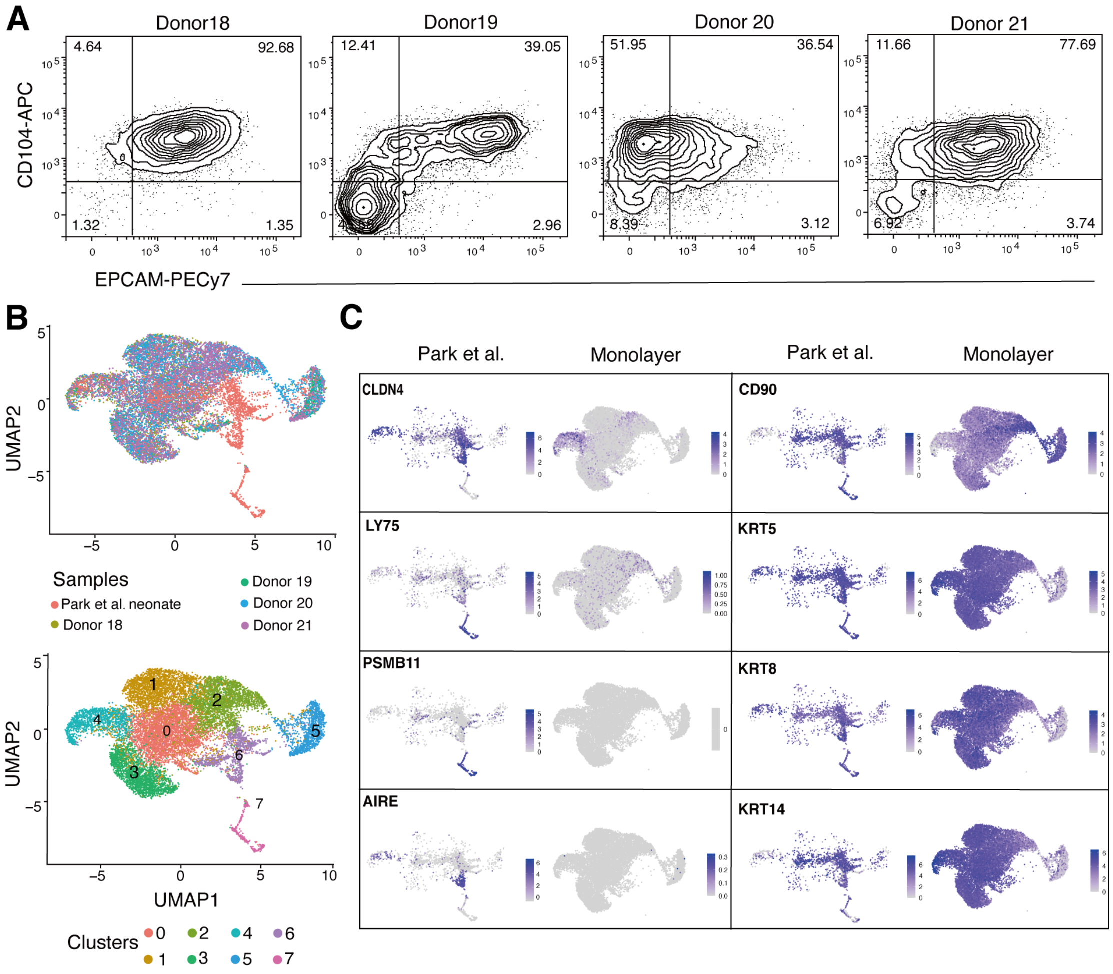

Figure S2 Single cell analysis of neonatal human thymus-derived monolayer cell cultures.

(A) Flow cytometry analysis for EPCAM and CD104 expression on neonatal human thymus-derived monolayer cells isolated from four independent donors (Donor 18-21). **(B)** Uniform Manifold Approximation and Projection (UMAP) analysis of single cell RNA-sequencing analysis showing cells from the four donors integrated with primary neonatal human thymic epithelial cells from the Human Thymus Cell Atlas (Park et al, 2020). Cells are clustered by samples used in data integration (upper) and cell type identities (lower). **(C)** Feature plots of integrated cells split into the primary human neonate thymic cells (Park et al.) and cultured monolayer cells (Monolayer). Gene features includes the TEC associated keratins (*KRT5*, *KRT8*, *KRT14*), the mTEC associated genes *CLDN4* and *AIRE*, the cTEC associated genes *LY75* and *PSMB11*, and *CD90*.

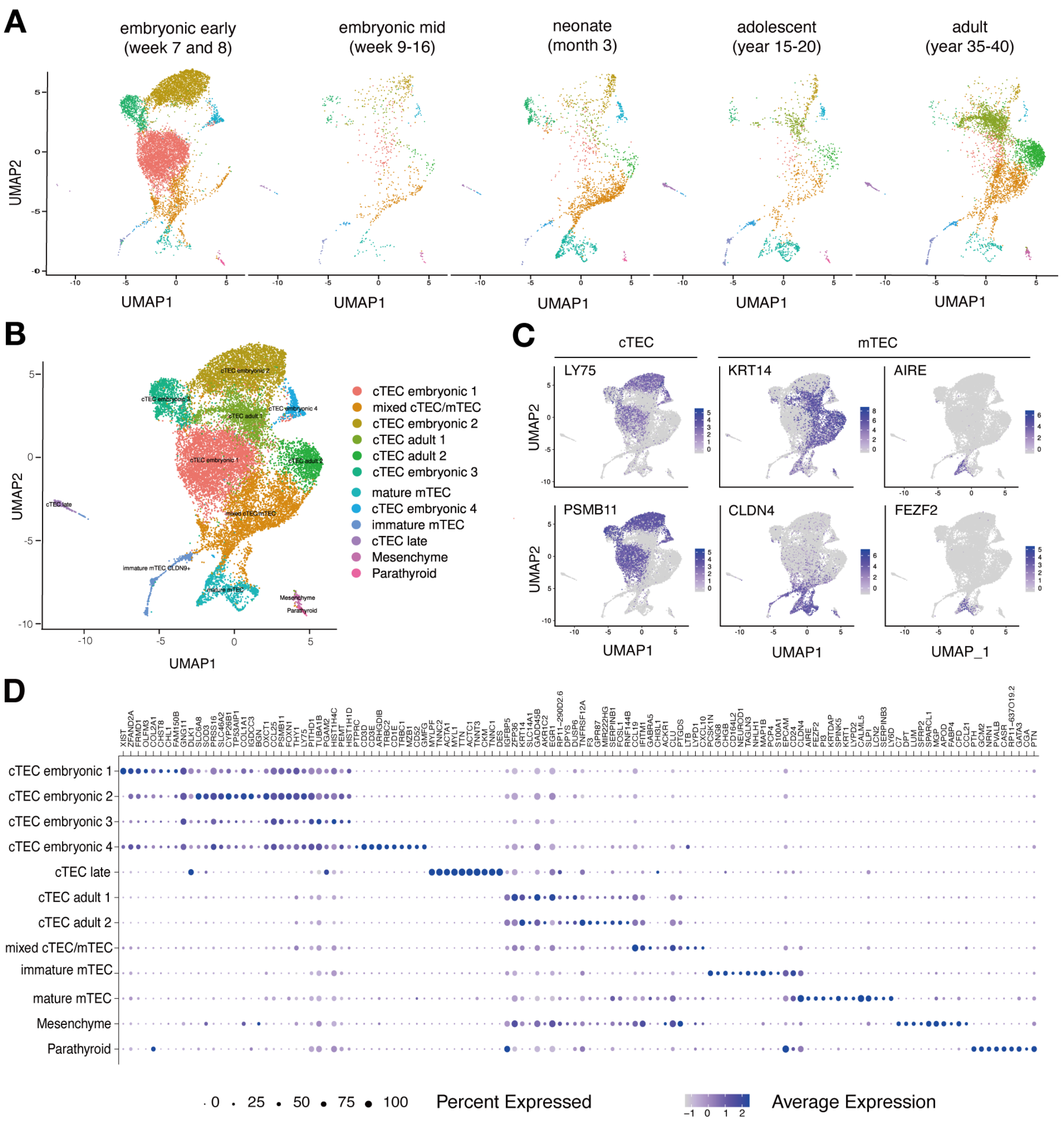

Figure S3. Single cell RNA-sequencing analysis of primary human thymic epithelial cells.

(A). Uniform Manifold Approximation and Projection (UMAP) analysis of single cell RNA-sequencing analysis of epithelial cells from the Human Thymus Cell Atlas split into development stages: embryonic early (week 7 and 8), embryonic mid (week 9 to 16), neonate (3 months), adolescent (year 15 to 20) and adult at time points (year 35 to 40). Cells are clustered and projected on UMAP plots. **(B)** UMAP projections of all human thymic epithelial cells grouped into clusters as indicated. **(C)** Feature plots showing the association between the expression levels of the indicated genes for cTEC populations (*LY75* and *PSMB11)* and mTEC populations (*KRT14, CLDN4, AIRE* and *FEZF2*). **(E)** Dot plot representation of cluster specific genes grouped into clusters described in (B**)**. The color intensity in each dot represents the average expression. Dot size represents the percentage of cells expressing that gene in its respective cluster.

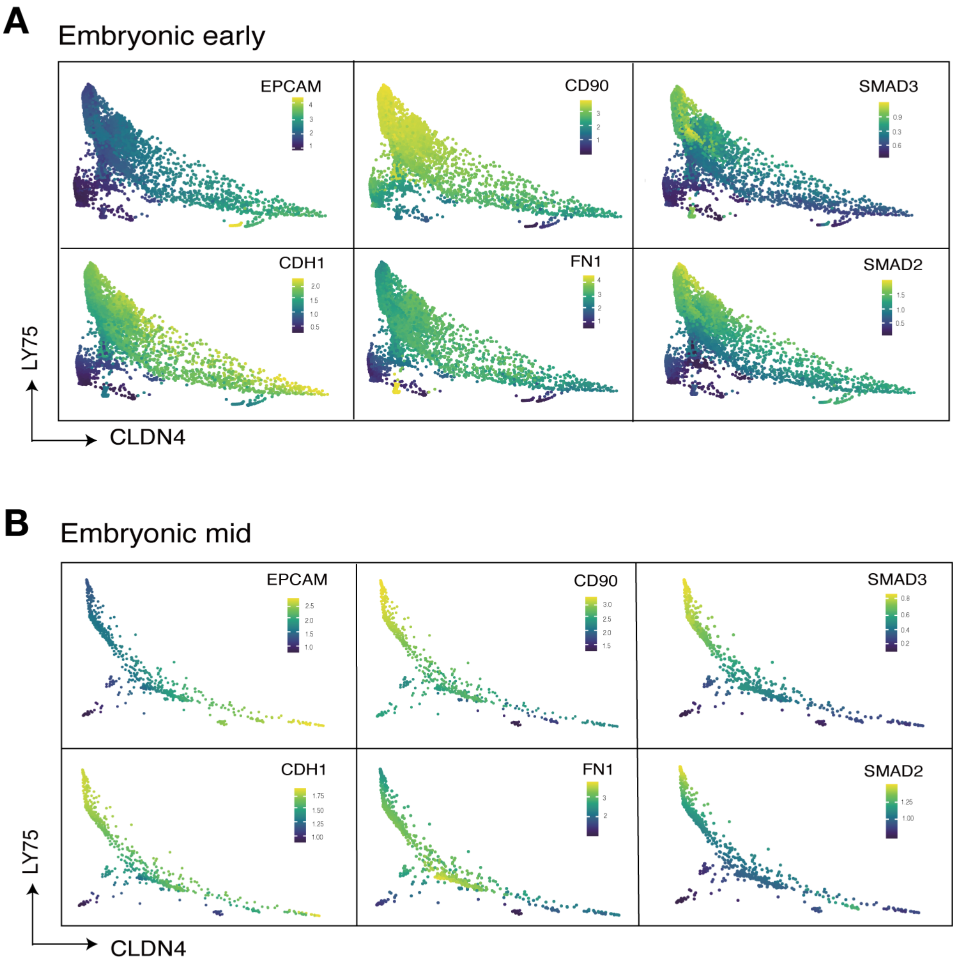

Figure S4. Association of epithelial/mesenchymal gene expression with cTECs and mTECs

Scatter plots showing the MAGIC imputed values calculated for EMT related genes that are expressed in TECs at the stage of embryonic early (week 7 and 8) **(A)** and embryonic mid (week 9 to 16) **(B)**. Each plot indicates the relative levels of gene expression for *EPCAM* and *CDH1*, representing an epithelial program, *CD90* and *FN1*, representing a mesenchymal program and *SMAD2* and *SMAD3*, representing genes involved in TGFβ signaling.

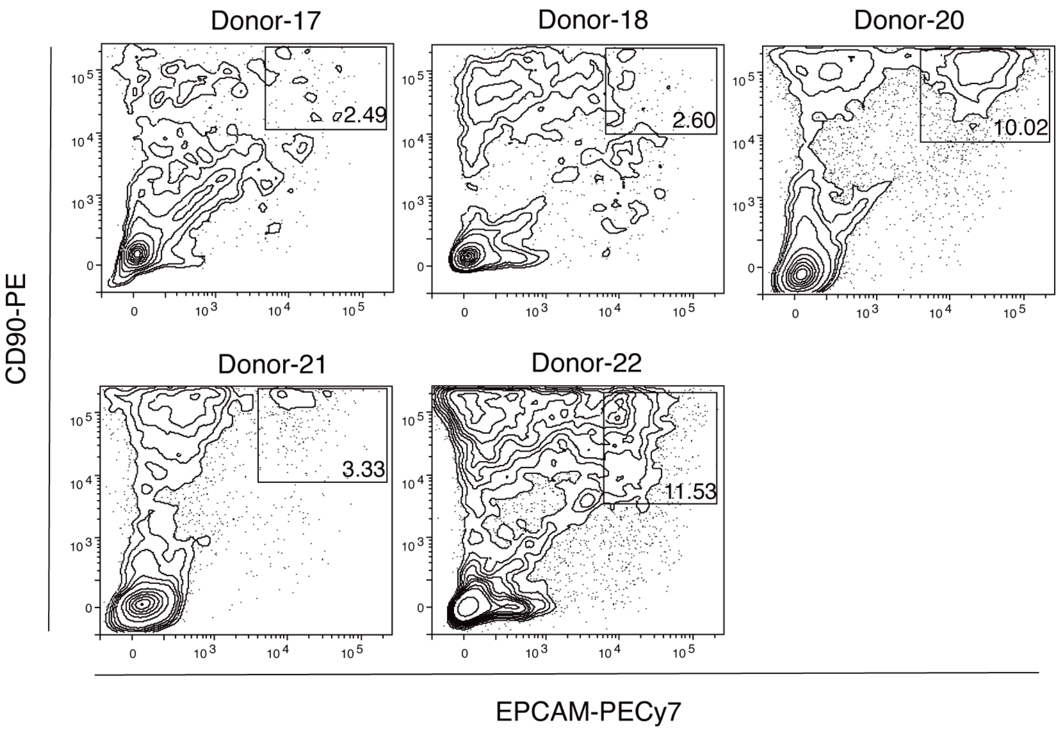

Figure S5 Flow cytometry analysis for EPCAM and CD90 expression in primary neonatal human TECs of additional five donors. *The* percentage of cells in the EPCAM+CD90+ gate is shown for each tissue donor (17-22). This data is summarized in **Figure 5**.

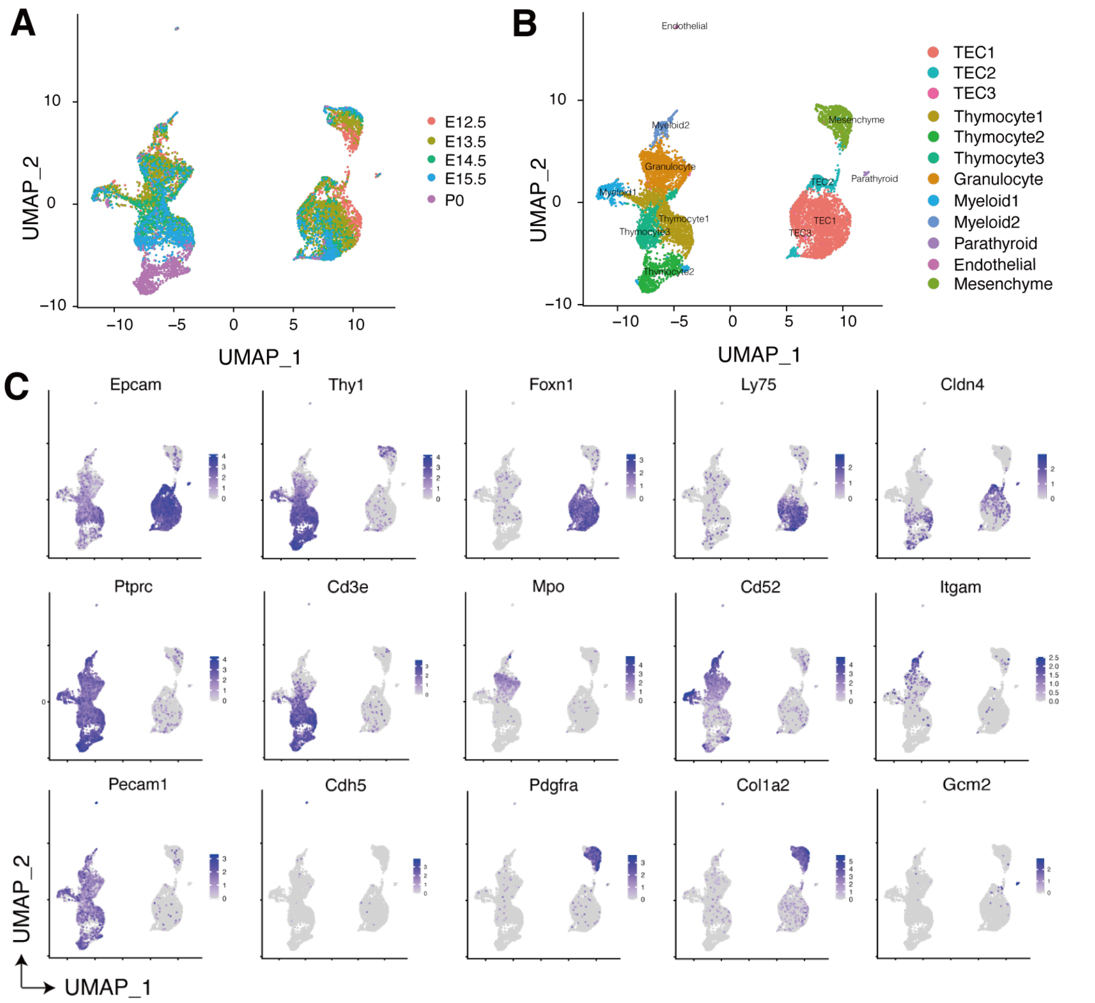

Figure S6 Single cell RNA-sequencing analysis of mouse embryonic thymic cells. **(A)**. Uniform Manifold Approximation and Projection (UMAP) analysis of single cell RNA-sequencing analysis of mouse thymic cells split into development stages (E12.5 – P0) (Kernfeld et al., 2018) **(B)** UMAP projections of all mouse thymic cells grouped into clusters with annotated cell identities as indicated. **(C)** Feature plots showing the expression levels of the indicated genes for the TECs (*Epcam, Thy1, Foxn1, Ly75* and *Cldn4*), thymocytes (*Ptprc, Thy1* and *Cd3e*), myeloid cells (*Ptprc, Mpo Cd52* and *Itgam*), endothelial cells (P*ecam1* and *Cdh5*), conventional mesenchymal cells (*Pdgfa* and *Col1a2*) and parathyroid cells (*Gcm2*).
